# Supplementary material for: Essential Fitness Repertoire of Staphylococcus aureus during Co-infection with Acinetobacter baumannii In Vivo
Source: mSystems. 2022 Aug 30;7(5):e00338-22. doi: 10.1128/msystems.00338-22 (PMC9600432; doi:10.1128/msystems.00338-22)
Supplement: TABLE S3 [file msystems.00338-22-s0003.docx]

| **Strains/plasmids** | **Characters** | | | **Source** |
| --- | --- | --- | --- | --- |
| **Strains** | |  | |  |
| S. aureus strain Newman | Background strain for Tn-seq library construction | | | Lab collection |
| S. aureus strain RN4220 | Restriction deﬁcient cloning host | | | Lab collection |
| A. baumannii strain ATCC19606 | Used as co-infection microorganism | | | Lab collection |
| E. coli strain Trans1-T1 | Cloning host for maintaining recombinant plasmids | | | Purchased from Transgen biotech |
| ΔsbnB | S. aureus strain Newman derivative with the sbnB gene replaced by a kanamycin resistance gene; KanaR | | | This work |
| ΔtreP | S. aureus strain Newman derivative with the treP gene replaced by a kanamycin resistance gene; KanaR | | | This work |
| ΔsasF | S. aureus strain Newman derivative with the sasF gene replaced by a kanamycin resistance gene; KanaR | | | This work |
| **Plasmids** |  | | |  |
| plasmid pMA15 | Plasmid containing a mariner transposon both with a kanamycin resistance gene and an MmeI restriction site within each inverted repeat , temperature sensitive, CmR | | | Collected in department of Laboratory Medicine, Ren Ji Hospital, School of Medicine, Shanghai Jiao Tong University |
| plasmid pBT2 | S. aureus-E. coli shuttle vector, temperature sensitive, AmpR in E. coli and CmR in S. aureus | | | Lab collection |
|  |  | |  | |

Supplemental Table 3. Strains and plasmids used in this study
