# Supplementary material for: Essential Fitness Repertoire of Staphylococcus aureus during Co-infection with Acinetobacter baumannii In Vivo
Source: mSystems. 2022 Aug 30;7(5):e00338-22. doi: 10.1128/msystems.00338-22 (PMC9600432; doi:10.1128/msystems.00338-22)
Supplement: TABLE S4 [file msystems.00338-22-s0004.docx]

Supplemental Table 4. Primers used in this study

| **Primers** | **DNA sequence from 5' to 3'** | **Function** |
| --- | --- | --- |
| OligoA | **5phos**/GATCGGAAGAGCACACGTCTGAACGCGAAGAT | Oligo that make up the adapter |
| OligoB | GTGACATCTTCGCGTTCAGACGTGTGCTCTTCCGATC NN | Oligo that make up the adapter |
| P1-transposon-MmeI*^a^* | XXXXXXXX XXXXXXXX AGACCGGGGACTTATCATCCAACCTGT | For amplification of a 103 bp Tn-seq fragment |
| P2-tn-seq-PCR*^a^* | XXXXXXXX XXXXXXXX GTTCAGACGTGTGCTCTTCCGATC | For amplification of a 103 bp Tn-seq fragment |
| sbnB-LAF | AGTGCAGCGGAATTCGAGCTCTTAAATTTGAGGAGGAAGCGC | For amplification of the left region of the sbnB gene |
| sbnB-LAR | CTAAAACCTTAAATCTGATTTAACTCCTTGTCTTGATTT | For amplification of the left region of the sbnB gene |
| sbnB-KaF | AGTTAAATCAGATTTAAGGTTTTAGAATGCAAGGAAC | For amplification of the KanR cassette |
| sbnB-KaR | CCTCGCATTCCTAAAACAATTCATCCAGTAAAATATAATATTTTATTTTCT | For amplification of the KanR cassette |
| sbnB-RAF | ATTGTTTTAGGAATGCGAGGTGTCTGAAC | For amplification of the right region of the sbnB gene |
| sbnB-RAR | CCTGCAGGTCGACTCTAGAGGATCCTCACAAACCATGACATACTTAGCT | For amplification of the right region of the sbnB gene |
| sbnB-LUF | AGCCTCCTTCGTGATGTATGAC | For identification of sbnB deletion |
| sbnB-RDR | CCATTGACACTAGCTGTTGCGT | For identification of sbnB deletion |
| treP-LAF | AGTGCAGCGGAATTCGAGCTCTAAAACGGATAGAAATCGAATCGTG | For amplification of the left region of the treP gene |
| treP-LAR | TAAAACCTTAAATCCCTTAATGTCTTAAAACGAGTATAAC | For amplification of the left region of the treP gene |
| treP-KaF | GACATTAAGGGATTTAAGGTTTTAGAATGCAAGGAAC | For amplification of the KanR cassette |
| treP-KaR | CTTTTTTATTTTACTAAAACAATTCATCCAGTAAAATATAATATTTTATTTTC | For amplification of the KanR cassette |
| treP-RAF | GAATTGTTTTAGTAAAATAAAAAAGGGGCGTTCGTTATT | For amplification of the right region of the treP gene |
| treP-RAR | CCTGCAGGTCGACTCTAGAGGATCCCGCCACCGTCATAAATACC | For amplification of the right region of the treP gene |
| treP-LUF | CAAATTTTAGAGCAAGGCGA | For identification of treP deletion |
| treP-RDR | TAAATATATGGCGTCCCTTGC | For identification of treP deletion |
| sasF-LAF | AGTGCAGCGGAATTCGAGCTCAAATAAGTATTATATTAAACCCGTAAAATTTATAAGTATAAAC | For amplification of the left region of the sasF gene |
| sasF-LAR | CTAAAACCTTAAATTATATTGGGGGAAGAGCATCT | For amplification of the left region of the sasF gene |
| sasF-KaF | CCCCCAATATAATTTAAGGTTTTAGAATGCAAGGAAC | For amplification of the KanR cassette |
| sasF-KaR | GGAGTATGTTGCTAAAACAATTCATCCAGTAAAATATAATATTTTATTTTCT | For amplification of the KanR cassette |
| sasF-RAF | AATTGTTTTAGCAACATACTCCTTCCTCACTTAC | For amplification of the right region of the sasF gene |
| sasF-RAR | CCTGCAGGTCGACTCTAGAGGATCCGCCCTACCATTTTCAGTGTTATATAA | For amplification of the right region of the sasF gene |
| sasF-LUF | GTGCAGATCAACATTACGGTC | For identification of sasF deletion |
| sasF-RDR | TCTGATGTCATATTGGATTGGC | For identification of sasF deletion |
| kana-InF | ACCGGAATTGAAAAAACTGAT | For identification of target gene deletion |
| kana-InR | CGCTTCTCCCAAGATCAATAA | For identification of target gene deletion |

^a^ The XXXXXXXX XXXXXXXX represents barcode sequence.
